# 17-Year-Old Boy with Renal Failure and the Highest Reported Creatinine in Pediatric Literature

**DOI:** 10.1155/2015/703960

**Published:** 2015-06-14

**Authors:** Vimal Master Sankar Raj, Jessica Garcia, Roberto Gordillo

**Affiliations:** ^1^Department of Pediatric Nephrology, University of Illinois College of Medicine at Peoria (UICOMP), Peoria, IL 61603, USA; ^2^Department of Pediatrics, University of Illinois College of Medicine at Peoria (UICOMP), Peoria, IL 61603, USA

## Abstract

The prevalence of chronic kidney disease (CKD) is on the rise and constitutes a major health burden across the world. Clinical presentations in early CKD are usually subtle. Awareness of the risk factors for CKD is important for early diagnosis and treatment to slow the progression of disease. We present a case report of a 17-year-old African American male who presented in a life threatening hypertensive emergency with renal failure and the highest reported serum creatinine in a pediatric patient. A brief discussion on CKD criteria, complications, and potential red flags for screening strategies is provided.

## 1. Background

Prevalence of chronic kidney disease (CKD) is increasing significantly and it has poor outcomes if not diagnosed and treated early in its course [1]. CKD is a public health issue that affects 9 to 12% of the population in the USA [2, 3]. When management is early and adequate, the rate of progression to kidney failure can be slowed, comorbidities prevented, and the morbidity and mortality of cardiovascular disease associated with CKD decreased. There is lack of information on incidence and prevalence of earlier stages of CKD in children, as most of these patients are asymptomatic [4].

The National Kidney Foundation (NKF) created the Kidney Disease Outcomes Quality Initiative (KDOQI) guidelines and definition of CKD to facilitate the diagnosis and management by primary care physicians. Serum creatinine, a product of muscle metabolism, has been widely used as a marker for glomerular filtration rate (GFR). We describe a clinical case of an adolescent male with the highest serum creatinine reported in a pediatric patient. The primary aim of this case report was to create awareness of chronic kidney disease among general practitioners and to stress that clinical manifestations could be subtle in the early stages of the disease.

## 2. Case Presentation

A 17-year-old African American male who was previously healthy with the exception of high blood pressure presented to a referring hospital with a 4-day history of coughing, vomiting, headache, facial edema, and lower extremity cramping. One day prior to admission, he also had noticed decreased urine output. At the referring hospital, he was found to be hypertensive with a serum creatinine of 52 mg/dL (4597 *µ*mol/L) and was transferred to our pediatric intensive care unit for further evaluation and treatment.

Patient was born at term gestation and there were no significant health problems. Patient during his routine clinic visits was noted to have high blood pressure by his primary care physician but no further evaluation was done as the blood pressure was attributed to his obesity. Patient did not have any prior surgeries and was not taking any medications. There was no significant history of renal disease, dialysis, or kidney transplant in any of the family members.

On admission, patient was noted to be afebrile, hypertensive with blood pressure of 179/93 mm Hg and heart rate of 88 beats per minute, and tachypneic with respiratory rate in the 40s but saturating at 100% on room air. His anthropometric measurements showed his height to be 180 cm, his weight to be 115 kg, and body mass index to be >99%. Pertinent positives in physical exam include obesity, respiratory distress with nasal flaring, bilateral periorbital edema, and bilateral lower extremity edema. Laboratory results showed the patient to have azotemia with blood urea nitrogen (BUN) of 203 mg/dL and serum creatinine of 52 mg/dL, measured by enzymatic method. He had severe metabolic acidosis with bicarbonate of 10 mmol/L. Patient also had severe electrolyte imbalances including hyponatremia (126 mmol/L), hyperkalemia (6.3 mmol/L), hyperphosphatemia (10.5 mg/dL), hypocalcemia (5.3 mg/dL), and severe anemia with hemoglobin at 5.1 g/dL. Parathyroid hormone levels were elevated at 682 pg/mL (normal 11–80 pg/mL). Urine dipstick showed 3+ protein and 2+ blood and a urine protein to creatinine ratio was elevated at 14 (normal <0.2). Serological workup including complement levels, antinuclear antibodies, antinuclear cytoplasmic antibodies, and antiphospholipid antibodies was negative. Chest X-ray was significant for cardiomegaly and pulmonary edema. Echocardiogram showed severe left ventricular hypertrophy. Renal ultrasound revealed bilateral small, echogenic kidneys (right kidney 7.7 × 5.2 × 4.8 cm; left kidney 8.7 × 4.3 × 4.1 cm) consistent with dysplastic kidneys. Patient emergently underwent hemodialysis for fluid overload and to correct electrolyte imbalance. His anemia and electrolyte imbalances were slowly corrected. He is currently transitioned to peritoneal dialysis awaiting kidney transplant.

## 3. Discussion

CKD is a heterogeneous group of diseases with altered structure and function of the kidneys with varying manifestation based on underlying etiology. Based on KDIGO guidelines, CKD is classified into various stages using GFR and the degree of albuminuria (Tables 1(a) and 1(b)).

The modified Schwartz formula used to calculate GFR is derived from the CKiD (chronic kidney disease in children) [5] and uses the following formula:

 eGFR = *K* (height in centimeters)/Serum creatinine with the constant *K* value being 0.413.

GFR varies with age, gender, and body size and increases from infancy to adulthood. Normative data of GFR based on age is presented in Table 2. This needs to be taken into consideration on CKD classification in pediatric population.

## 4. Epidemiology

Most epidemiological information on adult CKD is available from data on ESRD patients [6]. This represents only the tip of the iceberg and the actual incidence of early stage CKD will be much higher. The exact prevalence of childhood CKD is unknown but it has been estimated at 82 cases per million with ESRD around 15 cases per million based on national registries [7–9]. Though pediatric ESRD contributes only 2% to the total ESRD burden, the mortality rate in adolescents is about 30 to 150% higher compared to the general population [8, 10] indicating the need for specialized care. The expected remaining life time of children below 14 years of age with ESRD and on dialysis calculated per US renal data system (USRDS) [8] is only 20 years. Hence, the importance of primary prevention with early detection and aggressive intervention cannot be overstated.

## 5. Etiology

The etiology for ESRD varies with age. Structural anomalies contribute to a large degree to ESRD in younger children, while it is predominantly glomerular diseases in older children. As per NAPRTCS 2011 review data, about 14.2% of all pediatric dialysis patients had ESRD secondary to hypoplastic/dysplastic kidney with obstructive uropathy at 12.6% (Table 3).

## 6. Screening Strategies for CKD

Screening strategies are aimed at early detection and intervention for CKD so as to slow the progression of disease. Though routine urine screening for CKD has not been found to be cost effective in the general population and has not been recommended by AAP, it is important to identify and screen children at high risk. Some of the high risk populations at risk for CKD are mentioned below:

- Prematurity and being small for gestational age.
- Congenital abnormalities of the kidney and urinary tract.
- H/o poor growth or failure to thrive.
- Family history of kidney diseases and relatives on dialysis or transplant.
- Electrolyte or acid-base abnormalities.
- Body mass index (BMI) > 95th percentile.
- Blood pressure greater than the 95% recorded on multiple visits.
- Polyuria or inappropriately dilute urine.
- Gross hematuria.
- Dysfunctional voiding, urinary incontinence, or prolonged enuresis.
- H/o recurrent UTI.

A thorough history and physical examination during well child visits could help us in identifying these children with high risk for kidney disease. Once identified, these children should have their urine checked for proteinuria, their renal function analyzed by measuring creatinine, and their blood pressure regularly screened. Children with evidence of kidney damage should be sent to a specialist for further investigation and treatment. For our patient, blood pressure > 95% and the BMI > 95% should have prompted a screening for potential kidney disease.

## 7. CKD Complications

Cardiovascular disease accounts for most deaths in pediatric CKD similar to adult onset CKD. In contrast to adult CKD patients, where atherosclerosis and coronary vascular disease are much more common, arrhythmias account for the majority of cardiovascular death in children (19.6%) [11]. Traditional risk factors for CVD such as dyslipidemia and hypertension along with nontraditional risk factors such as anemia, disorders of calcium phosphorus metabolism, and increased chronic inflammation are highly prevalent in CKD population. These also contribute significantly to the cardiovascular burden in these children.

Anemia is a common complication in CKD secondary to impaired erythropoiesis. As CKD progresses, so does the prevalence of anemia in these children. Factors such as malnutrition, blood loss, iron deficiency, inadequate dialysis, and uncontrolled secondary hyperparathyroidism should always be kept in mind while managing resistant anemia. KDIGO recommends maintaining hemoglobin levels between 10 and 12 g/dL to reduce need for transfusion. Iron levels should be checked and adequately supplemented in all CKD patients before initiating or increasing dose of erythropoiesis stimulating agents.

Metabolic bone disorder (CKD-MBD) is a major complication in CKD. This occurs secondary to the inability of kidneys to excrete phosphorus and synthesize active vitamin D. Net result is secondary hyperparathyroidism. Disorders in calcium phosphorus balance and secondary hyperparathyroidism play a major role in vascular calcification in CKD and subsequent cardiovascular mortality and morbidity.

Fluid and electrolyte imbalances are especially common in children with CKD secondary to congenital anomalies of the kidneys and urinary tract (CAKUT). In CAKUT impaired urinary concentrating ability could present with polyuria and can present with dehydration. Disorder of sodium, potassium, and acid-base balance is also very common in these children due to impaired tubular handling. As the normal tubular response to ADH is not present, special attention should be paid to the fluid and electrolyte replacement in these children in the setting of dehydration.

Growth failure and neurocognitive delay are important concerns for young children with CKD [12, 13]. Providing adequate calories and protein intake are important in children with CKD as they are more prone to muscle wasting and anorexia. Factors such as systemic inflammation, oral aversion, and alteration in hormonal levels or resistance to action of hormones (follicle stimulating hormone, luteinizing hormone, growth hormone, and thyroid hormone) also contribute to short stature and CKD in children. In infants and toddlers with CKD to maximize nutritional intake a gastrostomy tube is often placed to provide adequate calories and fluids. Optimal nutrition and use of growth hormone replacement are often needed in children with CKD to ensure that they reach their growth potential.

## 8. Conclusion

Our patient had bilateral dysplastic kidneys and ultimately progressed to ESRD. Though he had potential red flags including obesity and high blood pressure noted on multiple occasions, the thought of kidney disease was not entertained. A screening urine dipstick could have prevented this life threatening admission with hypertensive emergency, severe anemia, and multiple electrolyte imbalances. Earlier detection of kidney disease and control of hypertension and proteinuria could have slowed the progression of disease and also would have allowed the time to plan for his renal replacement therapy in a safe manner. Chronic kidney disease is a growing health burden and awareness of it among primary care physicians is essential in early diagnosis and treatment.

## Figures and Tables

**Table:** (a)

GFR categories	Description	Range (mL/min/1.73 m^2^)
G1	Normal or high	≥90

G2	Mildly decreased	60–89

G3a	Mildly to moderately decreased	45–59

G3b	Moderately to severely decreased	30–44

G4	Severely decreased	15–29

G5	Kidney failure	<15

**Table:** (b)

Categories	Description	Range
A1	Normally to mildly increased	<30 mg/g

A2	Moderately increased	<3 mg/mmol 30–300 mg/g

A3	Severely increased	3–30 mg/mmol >300 mg/g

Adapted from KDIGO 2012.

**Table 2:** Normal glomerular filtration rate (GFR) in children and adolescents [15].

Age	Mean GFR ± SD (mL/min/1.73 m^2^)
1 week (males and females)	41 ± 15
2–8 weeks (males and females)	66 ± 25
>8 weeks (males and females)	96 ± 22
2–12 years (males and females)	133 ± 27
13–21 years (males)	140 ± 30
13–21 years (females)	126 ± 22

Adapted from National Kidney Foundation.

**Table 3:** Pediatric dialysis patient demographics [16].

All dialysis patients	*N*	%
	7039	100.0
*Primary diagnosis *		

FSGS	1016	14.4
Hypoplastic/dysplastic kidney	998	14.2
Obstructive uropathy	888	12.6
Reflux nephropathy	244	3.5
SLE nephritis	226	3.2

HUS	216	3.1
Chronic GN	214	3.0
Polycystic disease	201	2.9
Congenital nephrotic syndrome	182	2.6
Prune belly	144	2.0
Medullary cystic disease	140	2.0

Idiopathic crescentic GN	130	1.8

Familial nephritis	130	1.8

MPGN-type I	116	1.6

Pyelonephritis/interstitial nephritis	101	1.4

Cystinosis	99	1.4

Renal infarct	90	1.3

Berger's (IgA) nephritis	86	1.2

Henoch-Schönlein nephritis	67	1.0

MPGN-type II	64	0.9

Wilms' tumor	55	0.8

Wegener's granulomatosis	49	0.7

Drash syndrome	39	0.6

Other systemic immunologic diseases	37	0.5

Oxalosis	32	0.5

Membranous nephropathy	29	0.4

Sickle cell nephropathy	21	0.3

Diabetic GN	10	0.1

Other	887	12.6

Unknown	528	7.5

Adapted from NAPRTCS 2011.

## Abbreviations

CKD: Chronic kidney disease
ESRD: End stage renal disease
GFR: Glomerular filtration rate
BUN: Blood urea nitrogen.
